# The process of transition from pediatric to adult healthcare services for nephrological patients: Recommendations vs. reality—A single center experience

**DOI:** 10.3389/fped.2022.954641

**Published:** 2022-08-23

**Authors:** Dorella Scarponi, Gabriella Cangini, Andrea Pasini, Claudio La Scola, Francesca Mencarelli, Cristina Bertulli, Domenico Amabile, Marco Busutti, Gaetano La Manna, Andrea Pession

**Affiliations:** ^1^Pediatric Unit, IRCCS Azienda Ospedaliero-Universitaria di Bologna, Bologna, Italy; ^2^Department of Specialist, Diagnostic and Experimental Medicine, Alma Mater Studiorum University of Bologna, Bologna, Italy; ^3^Specialty School of Pediatrics, Alma Mater Studiorum University of Bologna, Bologna, Italy; ^4^Nephrology, Dialysis and Renal Transplantation Unit, IRCCS Azienda Ospedaliero-Universitaria di Bologna, Bologna, Italy

**Keywords:** transitional care, CKD, adolescents, health-related quality of life, disease awareness, self-management skills

## Abstract

Transitional care is an essential step for patients with kidney disease, and it is supported by policy documents in the United Kingdom and United States. We have previously described the heterogeneous situation currently found in Europe regarding certain aspects of transitional care: the written transition plan, the educational program, the timing of transfer to adult services, the presence of a coordinator and a dedicated off-site transition clinic. In line with the transition protocol “RISE to transition,” the objective of this paper is to describe the experience of the Bologna center in defining a protocol for the management of chronic kidney disease and the difficulties encountered in implementing it. We apply this model to various chronic diseases along the process of transfer to adult services. It begins when the patient is 14 years old and is complete by the time they reach 18. The family is continuously involved and all the patients in transitional care receive continuous medical care and psychological support. We identified a series of tests designed to measure various criteria: medical condition, psychological state, quality of life, and degree of patient satisfaction, which are repeated at set intervals during the transition process. The organization of the service provided an adequate setting for taking charge of the patients in the long term. The transition program implemented by the adult and pediatric nephrology services of the Bologna center has lowered the risk of discontinuity of care and greatly improved the patients’ awareness of responsibility for their own healthy lifestyle choices.

## Introduction

The number of young patients transitioning from pediatric to adult renal care has been progressively increasing for some time, mainly due to improvements in antenatal screening for nephrourological anomalies, which have resulted in a growing number of children with chronic kidney disease (CKD) being followed by pediatric nephrologists, and improved management of acute kidney injury with patient survival rates of 85–90% (1–3). From a healthcare perspective, the transfer between different contexts of care (different approaches to the same disease are often seen in pediatric and adult nephrology units) should ideally be a gradual process within a care pathway guaranteeing, on the one hand, coordination and continuity of care and, on the other, the sustained treatment adherence of each adolescent (4). Therefore, such a program should include a revision of logistic and organizational, but also educational, aspects in order to motivate transfer and make sense of the transition experience for all concerned (patients, family members, healthcare professionals) in a simple “handover.”

Due to their complexity, the pediatric patients under our care require a dynamic care pathway, from diagnosis, which accounts for the different stages of growth and development and is therefore adaptable according to the patient’s age, physical, cognitive, psychological, and social skills, as well as the course of their renal disease *per se*.

The transition process is therefore a reference framework within which the patients, their families, and the healthcare professionals prepare for the passage from adolescence to early adulthood, and from the pediatric to the adult service. Such a model requires healthcare professionals to adopt a more open form of communication, an educational attitude toward the understanding and management of the disease and its treatment by the patient-parent, and an introduction to the adult service (5).

In order to be effective, this process should not begin just before the patient’s eighteenth birthday, but well before so as to actively involve youths in their own care pathway (6).

In fact, it is well known that when the transfer from one context of care (pediatric) to another (adult) occurs without adequate prior communication it can cause a sense of disorientation and confusion in patients, which can reduce treatment adherence at the adult service and accelerate dropout (7). As patients will be required to attend less regular visits at the adult unit, an increased risk of clinical and prognostic worsening may be seen (8).

As parents’ disease knowledge influences that of their adolescent children, family members must be involved in these phases in order to receive support for encouraging growing disease awareness in their children, favoring adaptive behavior, and facilitating their autonomy in managing their disease (taking medications, booking appointments, etc.) (9). Parents should participate in the educational processes proposed by the clinicians and gradually reduce their involvement in the management of the disease, especially once the patient has reached adulthood (10).

The personnel involved in the transition process must work in close collaboration, and plan a series of meetings before the moment of transfer to promote their mutual knowledge of the patient, from a clinical and relational standpoint. Furthermore, a series of logistical information (who will be responsible for the patient, where the adult service is located, and the relevant contact details) must be supplied and organizational arrangements made in order to identify the most suitable out-patient clinic for each case (11).

As clinicians, we must expect some degree of failure in the transition process, as reported in the literature (12). For this reason, the progress of the process must be monitored by means of accurate reporting of the number of transitioned patients, the number of dropouts, and through sharing the first-hand experiences of the patients and their care-givers, also in terms of their perceived quality of life, even as early as the first follow-up visit after transition.

## Description of the process

Transitional care is an essential step for our patients with kidney disease. We have previously described the heterogeneous situation currently found in Europe regarding certain aspects of transitional care: the written transition plan, educational programs, the timing of transfer to adult services, the presence of a coordinator and a dedicated off-site transition clinic (13). In order to address the problem of heterogeneity in transitional care models, we have developed an interventional model designed around the available healthcare resources of our hospital.

In line with the transition protocol “RISE to transition,” which can be considered a milestone, the Bologna center wants to share its experience in defining a protocol for the management of CKD, and the difficulties encountered in implementing it (14).

We selected personnel from the pediatric and adult services and formulated the transition plan together. The clinicians met to identify and establish the different roles within the team as well as the care plan and its organizational-management aspects (dedicated spaces, timing). The two multidisciplinary structures share the same objectives: the inclusion and empowerment of the patients, continuity of care, the integration of different healthcare interventions. This process was facilitated by the fact that the two services are located near to each other on the hospital grounds.

We chose a pediatric nephrologist as the coordinator responsible for organizing the transition plans for all the patients because they interact with the patient and the family from the beginning of the clinical history, which means they have a full understanding of the patient, their disease, their prognosis, and can therefore provide the psychologist with valid suggestions for finding the best approach. The pediatric nephrologist is the person who interacts with all the other specialists (ophthalmologist, orthopedic doctor, etc.) and healthcare professionals (dieticians, physiotherapists) and therefore has a global understanding of the patient’s problems. Thanks to the “imprinting” that is established with the patient and their family they are the professionals who can, more than any other, gradually begin to give the patients information about their disease and initiate detachment from their parents (7). The coordinator works in harmony with the transition team, which comprises all the pediatric nephrologists in the unit, a medical psycho-physiologist, a nurse, and a social worker. It is also possible to consult other specialists if necessary.

Furthermore, our Pediatric Nephrology and Dialysis Unit is the regional reference center for nephrological diseases in children, which means that patients diagnosed at other centers in the Emilia Romagna region may also be included. The project was formally initiated in 2020.

## Study population and inclusion criteria

Given that the number of young patients graduating from pediatric to adult renal care has progressively increased, it was important to clearly define which patients, among those with chronic nephrological diseases, had to be included in our transition process. To date, we have included 83 patients and their families.

Severity of the disease was the first criteria adopted: all kidney transplant recipients (13/83 patients; 15%) and patients with CKD in at least stages II-III (23 pts; 28%), regardless of their primary etiology, were included. We further included all patients with kidney disease at risk of progression toward CKD: chronic glomerulopathies such as systemic lupus erythematosus, membranous glomerulonephritis, C3 glomerulonephritis, vasculitis, etc. (13 pts; 15%) and nephrotic syndrome (11 pts; 13%), genetic diseases, such as atypical hemolytic uremic syndrome (6 pts; 7%), autosomal recessive or dominant polycystic kidney disease and Alport syndrome (9 pts; 11%), tubulopathies (Dent syndrome, Bartter syndrome, etc.), hyperoxaluria, cystinuria, congenital anomalies of the kidney and urinary tract with severe renal dysplasia (9 pts; 11%).

Of note, six of the patients included had syndromes involving the kidney and causing reduced autonomy (Lowe syndrome, tuberous sclerosis, Bardet Biedle syndrome, etc.); these patients are unable to express their ideas autonomously once they reach adulthood, however, they still require more personalized transitional care. We accounted for all of the clinical situations that significantly affect the psycho-neurobehavioral status of the patients and for which it is necessary to adjust the pathway, for example a longer transition time or transition accompanied by a caregiver, without any limitations to the healthcare they receive (7). The COVID-19 pandemic caused reduced and delayed access to out-patient clinics for chronic patients, thus decreasing the activity of the program, which resumed normal activity in 2021. Recently, the transition of 45 new 14-year-old patients with different kidney diseases was initiated; 17 of them have already reached 16 years of age and have therefore met the adult team together with the pediatricians.

## Timing of the process

The process of transfer to adult services begins when the patient is 14 and is complete by the time they reach 18 years of age. During this period, all the patients in transitional care receive continuous medical care and psychological support and their families are involved throughout the entire process. At the beginning of the transition process, all patients are offered a clinical psychology interview, including psychometric assessment. The psychological evaluation consists in a series of clinical interviews in support of the patient and their family focused on managing the difficulties they have in coping with the disease. In addition, we identified a series of tests designed to measure various criteria such as depression and anxiety, quality of life, degree of patient satisfaction, all significantly affected by the status of chronic disease in adolescence (15). These evaluations are repeated at set intervals during the transition process (Table 1).

**TABLE 1:** Outline of the stages of the transition process from pediatric to adult healthcare.

Transition process			
When	14 years of age	16 years of age	18 years of age
Where	PEDIATRIC NEPHROLOGY Clinic	TRANSITION Clinic	ADULT NEPHROLOGY Clinic
What	MEDICAL VISIT INTERVIEW WITH PEDIATRIC TEAM PSYCHOLOGICAL ASSESSMENT: • Psychological interview • Tests: Children’s depression inventory (CDI), anxiety scale (Busnelli-Dall’Aglio-Farina)	MEDICAL VISIT INTERVIEW WITH PEDIATRIC AND ADULT TEAM PSYCHOLOGICAL ASSESSMENT: • Psychological interview • Test: Beck depression inventory (BDI-II), the state-trait anxiety inventory (STAI-Y)	MEDICAL VISIT INTERVIEW WITH ADULT TEAM PSYCHOLOGICAL ASSESSMENT: • Psychological interview, • Test: BDI-II, STAI-Y, QV
Who	Pediatric team	Pediatric and adult team	Adult team
For whom	PATIENT with PARENTS/CAREGIVERS	PATIENT with PARENTS/CAREGIVERS	PATIENT and PARENTS/CAREGIVERS

When a patient reaches 14 years of age, the doctor and the psychologist inform them and their family about the transition process, the healthcare professionals involved, and the medical appointments they will need to attend. These moments, during which the medical personnel interact with the patient and their family, provide an opportunity to begin investing in the patient as a person who is potentially capable of increasing autonomy. We offer the patient a psychometric evaluation, which involves the administration of age-appropriate tests aimed at assessing anxiety and mood levels (Table 2). The results, which are discussed with the patient and their family, may indicate the need for an actual psychotherapeutic intervention (16).

**TABLE 2:** Psychometric tests administered during the transition process from pediatric to adult healthcare.

Transition process psychometric tests			
Age	14 years	16 years	18 years
Depression	CDI Children’s depression inventory	BDI-II Beck depression inventory-II	BDI-II Beck depression inventory-II
Anxiety	BUSNELLI Anxiety scale questionnaire for evolutive age	STAI-Y State-trait anxiety inventory Y	STAI Y State-trait anxiety inventory Y
Quality of life PATIENTS	/	/	PedsQL Pediatric quality of life inventory version 4.0 generic core scales
Quality of life PARENTS/CAREGIVERS	/	/	PedsQL Pediatric quality of life inventory version 4.0 generic core scales

The clinical psychology interviews take place between 14 and 16 years of age, providing the educational support necessary for encouraging the development of each family unit’s compliance and resilience skills. Their main objective is to reduce the fear of the unknown expressed by adolescent patients and to increase confidence and trust in a new medical team (17).

When the patient reaches 16 years of age, a multidisciplinary appointment is organized during which the pediatric team introduces the patient to the adult nephrologist and psychologist responsible for the transition process. In this way it is possible to create an initial shared space, where everyone speaks the same professional language in order to give the family a sense of continuity of care. This set-up remains the same for every follow-up visit until the patient turns 18.

The psychological interviews which take place between 16 and 18 years of age include psychometric evaluation, which is repeated in cases where significant psychological suffering has been previously diagnosed and when the patient has undergone psychotherapy and it is necessary to assess its efficacy in terms of psychological health (Table 2).

Once the patient is 18, at the end of the clinical appointment in the transition clinic, and depending on the disease and/or CKD stage, the patient and their caregivers are provided with the necessary information regarding their first appointment at the adult Nephrology, Dialysis and Transplant Unit. As the adult nephrology unit is located near the pediatric unit, the discomfort linked to a change in settings which are culturally very different is greatly reduced (18).

The adult nephrologist in the transition team initially takes charge of the patient, irrespective of their type of kidney disease. Only subsequently will the patient be assigned to the appropriate out-patient clinic based on their specific nephropathy (genetic diseases, glomerular diseases, CKD, dialysis, transplantation). The initial mode of access to the adult unit is therefore identical for all patients: the date and time of the clinical visit and blood tests is provided at the end of the final appointment in the transition clinic. The timing of the first adult appointment is dictated by the clinical condition of the patient at the moment of transition. In the case of patients who request transfer to an adult nephrology unit at a different hospital, the adult team will organize a meeting with the receiving hospital team to guarantee continuity of care.

Within the first year after transfer to the adult service, the patient is asked to undergo a psychological-psychometric evaluation. The psychometric tools adopted will guide the possible psychological support in keeping with any potential psychological emergencies, even in adulthood.

This initial psychological follow up involves the psychological re-assessment of the patient to which the evaluation of the quality of life perceived by the patient and their caregivers is added (Table 2). This last assessment guides the transition program and its efficacy (19).

## The process in real life

The transition process was defined, in organizational terms, in accordance with the hospital services and, after a careful review of the literature, a model that is currently in line with the available staffing, logistic, and cultural resources was created. The participating specialists were identified and began planning the transition clinic, a dedicated setting in the pediatric unit. It was necessary to set up the activities of this clinic in parallel with the regular out-patient clinic activities, and on a different time schedule in order to facilitate participation by the multidisciplinary team members. The clinic was set up on a weekly basis. Specific codes identifying the services provided within the transition pathway were established and a shared, personalized electronic patient file was created containing the medical reports submitted by the different specialists involved (6). Therefore, the entire transition history of each patient is documented and immediately consultable by each member of the team (20).

## Difficulties encountered and solutions found

We encountered several difficulties during the initial stages. For example, we were met with some resistance from the hospital regarding the proposed changes to the management of these patients, which we overcame by meeting with hospital administrative staff to discuss, plan and create the organizational model. The problems caused by differences in the pediatric and adult models of care were resolved by selecting a pediatric team and an adult team who participated in joint meetings and training sessions. We reinforced professional networks to promote continuity of care between centers for patients who transfer to an adult unit in a different hospital or city. We contacted and established relationships with local mental health facilities in the residential areas of patients with cognitive deficits, and ensured continuity between the hospital social services and those on the territory for families with social problems. Educational therapy provided support for families when passing from the pediatric to the adult reality, while psychological evaluation and interviews provided clarification and support to patients as they gradually become more autonomous and less dependent on their families. The face-to-face discussions and consultations in the pediatric unit were conducted in order to identify the best communicative approach necessary for establishing a personalized transition support intervention program.

## Adherence to the transition process

Although the pathway is relatively new, we have seen good adherence in such that the families have continually expressed interest in the process, mainly because they were involved well in advance, and appreciation for the educational approach to the whole family adopted by the team. The main difficulty recognized concerned the passage from family-based consultations to adolescent-focused care (21). Nonetheless, the caregivers feel that they have broadened their parent-child relational perspective, recognizing ever-improving self-management skills in their children. The patients were pleasantly surprised by an approach which recognizes their growing autonomy, but were fearful of being accountable for their own health. The patients and their parents requested interviews with the psychologist, both individually and as a group, even after transfer, which is an indication of the trust which developed between the families and the staff and the mutual respect within the families. In order to minimize the difficulties the staff had in finding the correct approach to use with both the families and the adolescents, and to improve their communication style, specific meetings were introduced into the annual training schedule of the dedicated pediatric staff. Various meetings were also held with the adult nephrology team, with the aim of creating a stable network of professionals with the skills necessary for dealing with the transition process. To date, we have had no dropouts.

## Discussion

This paper gives a detailed account of the transition process for patients with kidney disease in our hospital in Bologna, Italy. In our experience, the organization of the transition service, based on the recommendations compiled by the multidisciplinary team and approved by the Parents’ Association, has provided an adequate setting for taking charge of the patients in the long term.

The level of satisfaction among patients and caregivers is high, as reflected by the fact that there have been no dropouts so far. The most recognized key points in the process of assessment and accompaniment of the patient in terms of their healthcare-related aspects are the presence of a coordinator, the timing of transfer, the logistics, and the multidisciplinarity of the team. The biggest limitations capable of reducing the applicability of the model in our experience are those clinical situations which require the collaboration of highly specialized professionals not included in the multidisciplinary team (e.g., professionals skilled in communicating with deaf, blind and mute patients), a limited number of dedicated resources in relation to the growing number of patients, and the poor medication compliance of some patients and/or their caregivers.

In assessing the efficacy of the transition program implemented by the adult and pediatric nephrology services of the Bologna center, it is evident that the risk of discontinuity of care has decreased and the patients’ awareness of and responsibility for their own healthy lifestyle choices has greatly improved. The implementation of this model aims to improve adherence rates, rejection rates, and quality of life. The results of our study may serve as a basis for further research, which could contribute to further improving the transition experience for pediatric renal patients in Italy.

## Data availability statement

The original contributions presented in this study are included in the article/supplementary material, further inquiries can be directed to the corresponding author.

## Author contributions

DS and GC contributed to the conception and design of the work and wrote the first draft of the manuscript. APa, GL, and APe contributed to the conception and design of the work and critically reviewed the manuscript. CB, CL, FM, DA, and MB critically reviewed the manuscript. All authors contributed to manuscript revision, read, and approved the submitted version.
